# Ultrasound-Guided Abrams Pleural Biopsy vs CT-Guided Tru-Cut Pleural Biopsy in Malignant Pleural Disease, a 3-Year Follow-up Study

**DOI:** 10.1007/s00408-016-9933-9

**Published:** 2016-08-19

**Authors:** Parthipan Sivakumar, Deepak Jayaram, Deepak Rao, Vignesh Dhileepan, Irfan Ahmed, Liju Ahmed

**Affiliations:** 1 Kings College London, London, WC2R 2LS UK; 2 East Surrey Hospital, Redhill, RH1 5RH UK; 3 Princess Royal University Hospital, Orpington, UK; 4 Guy’s and St Thomas’ NHS Foundation Trust, London, UK

**Keywords:** Pleura, Malignancy, Biopsy, Abrams, Tru-Cut, Biopsy

## Abstract

**Purpose:**

Conventional Abrams biopsy shows low sensitivity in suspected malignant pleural disease. There are limited data on the improvement in sensitivity by adding in image guidance. This retrospective study compares the diagnostic sensitivity of Abrams biopsy using ultrasound guidance with CT-guided Tru-Cut biopsy in suspected malignant pleural disease.

**Methods:**

Data were collected from 2006 to 2012 of patients who underwent image-guided biopsies for suspected non-tuberculous pleural disease. Data were collected on the result of the initial biopsy and final patient diagnosis as of June 2015.

**Results:**

Sixty-three patients underwent image-guided Abrams biopsy and 29 underwent CT-guided Tru-Cut biopsies. The sensitivity of Abrams was 71.43 % compared to 75 % in the CT-guided Tru-Cut group. Specificity was 100 % in both groups.

**Conclusions:**

Image-guided Abrams biopsies demonstrate comparable diagnostic sensitivity in malignant pleural disease to CT-guided Tru-Cut biopsy.

**Electronic supplementary material:**

The online version of this article (doi:10.1007/s00408-016-9933-9) contains supplementary material, which is available to authorized users.

## Introduction

There are more than sixty recognised causes for pleural effusion. Pleural fluid biochemistry and cytology are often the first invasive test. In suspected malignant pleural disease, diagnostic thoracocentesis offers a positive cytological diagnosis in 50–60 % of cases [1], with a sequential gain of 27–31 % from a repeat procedure [2]. If pleural fluid cytology is negative, histology is necessary to establish the diagnosis. Thoracoscopic pleural biopsy remains the gold standard with a diagnostic yield of up to 99 % [3–5]. However, patients need to be sufficiently fit enough to undergo either video-assisted thoracoscopic surgery or local anaesthetic “medical” thoracoscopy. Percutaneous pleural biopsy is a less invasive alternative, particularly indicated when pleural malignancy or tuberculosis is suspected.

Historically closed pleural biopsies are mostly performed using an Abrams needle or Tru-Cut needle. Abrams needles are reversed bevelled punch biopsy needles that allow tissue sampling to be performed as a day case in a dedicated procedural area or at the bedside depending on local practice. When performed in the traditional sense as a “blind” procedure, the diagnostic sensitivity in malignant disease is variable (between 40 and 73 % [6–8]). Although real-time visualisation with ultrasound (US) whilst using Abrams needle is not possible, it can be used to direct site selection during the procedure, increasing yield to 60–77.4 % [9]. Incorporating CT guidance to target areas of pleural disease has been shown to increase the sensitivity to 87.5 % [10].

Percutaneous CT-guided Tru-Cut cutting needle biopsies are considered a superior diagnostic method. These are typically performed by radiologists. The diagnostic sensitivity in malignant disease is consistently superior to blind Abrams biopsy at approximately 87 % [6, 11].

There are no studies comparing ultrasound-guided Abrams to CT-guided Tru-Cut biopsies in malignant pleural disease. We hypothesise that ultrasound-directed percutaneous Abrams biopsies will produce comparable results to CT-guided Tru-Cut biopsy. We retrospectively evaluated both approaches, following up patients over 3 years to assess the sensitivity of these diagnostic modalities in suspected malignant disease.

## Materials and Methods

As a retrospective service evaluation, written patient informed consent and regional ethics approval were not required. Local clinical governance committee approval was needed for the use of patient records, which was obtained.

Using radiology and pleural clinic databases, we identified all patients who underwent a CT-guided Tru-Cut pleural biopsy or US-guided Abrams pleural biopsy for suspected non-tuberculous pleural disease with no cytological diagnosis on previous fluid aspirate from February 2005 to September 2012 at Guys and St Thomas’ NHS Foundation Trust. We reviewed hospital inpatient documentation, radiological reports, clinic letters, cytological, and histological results. All procedures were performed on an outpatient basis, unless the patient had already been admitted to hospital. Patients with suspected or proven granulomatous disease were excluded. Suspicion of granulomatous disease was defined as clinical and/or radiological features highly suggestive of pulmonary or extrapulmonary tuberculosis in the absence of positive microbiological confirmation at the time of pleural biopsy.

### Abrams Biopsy

This is performed in a designated area in an outpatient setting by a trained and experienced consultant and supervised specialist registrars. Ultrasound image guidance is employed to identify sites of pleural abnormality. Under local anaesthesia, closed pleural biopsies are then performed using a reverse-bevelled Abram biopsy needle.

### CT-Guided Tru-Cut Needle Biopsy

Cases are vetted at radiology meetings based on their amenability to biopsy. These are performed under local anaesthesia by trained interventional radiologists. CT is used to directly visualise and target the biopsy site with a Tru-Cut cutting needle.

We recorded the histological result of the initial biopsy in both groups. The standard employed to determine diagnostic accuracy was a final diagnosis of mesothelioma or metastatic pleural malignancy made up to September 2015 or at death (whichever came sooner). If a patient died within the follow-up period, clinical notes were reviewed to ascertain if a cancer diagnosis was made at the time of death. The volume of biopsy specimens was attained from the histology reports. Data on subsequent pleural procedures were also recorded.

Statistical analyses were performed using IBM SPSS version 22. Continuous data are summarised as mean with standard deviation (for normally distributed data) or median with 25–75 % interquartile range (for data with a skewed distribution). Binary data are presented as percentages. The Mann–Whitney *U* test was used for variables with skewed distribution.

## Results

### Diagnostic Utility

During the study period 63 patients underwent Abrams biopsy and 29 patients underwent CT-guided biopsy for suspected non-tuberculous pleural disease (Table 1). Final cancer diagnoses are available in supplementary table S1.

**Table 1:** Patient demographics and diagnostic yield in the two groups

	Ultrasound-guided Abrams group	CT-guided Tru-Cut group	
Number of patients	63	29	
Male	39 (61.9 %)	19 (65.5 %)	Chi square *p* = 0.739
Age (years), mean (SD)	64.4 (15.97)	69 (15.22)	Independent t *p* = 0.196
Diagnosis on initial biopsy			Chi square *p* = 0.326
Malignancy	25 (39.7 %)	15 (51.7 %)	
Benign/alternative diagnosis	36 (57.1 %)	12 (41.4 %)	
Non-diagnostic/no pleural tissue	2 (3.0 %)	2 (6.9 %)	
Final diagnosis at 3 years			Chi square *p* = 0.316
Malignancy	33 (+2 clinical diagnoses) (55.6 %)	22 (75.9 %)	
Benign	28 (44.4 %)	6 (20.7 %)	
Unknown	–	1* (3.4 %)	
Overall sensitivity	71.43 %	75 %	

* This patient was discharged to a nursing home following the biopsy and lost to further follow-up

**Fig. 1:**
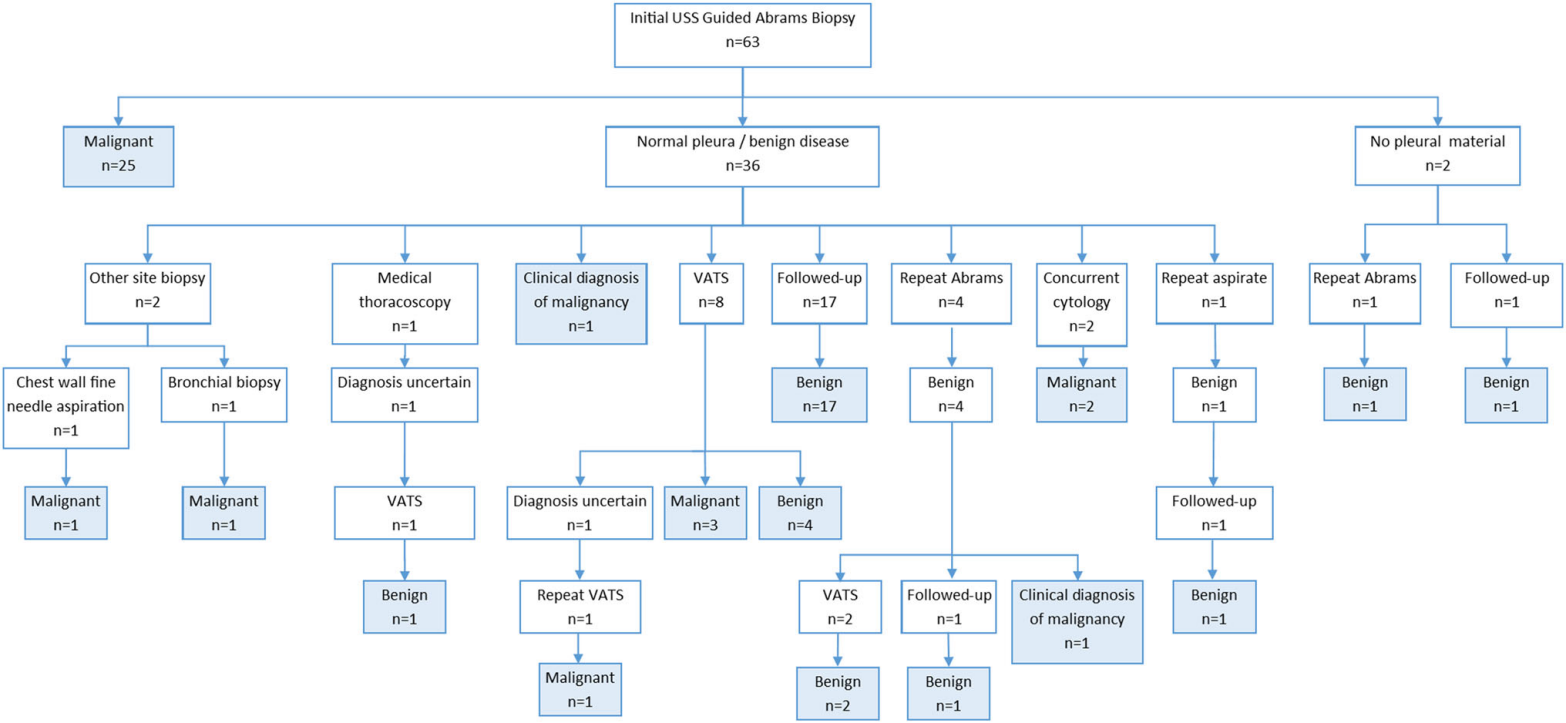
Diagnostic pathway of the patients in the US-guided Abrams biopsy group. *Shaded boxes* represent a diagnosis of malignant or benign disease at the end of the follow-up period (*VATS* video-assisted thoracoscopic surgery)

In the US Abrams group (Fig. 1) , malignancy was identified in 25 (39.6 %) patients from the initial biopsy. Biopsies were non-diagnostic (no pleural tissue) in two cases. The remaining 36 (57.1 %) had either normal histology or was consistent with a benign process. One of these patients was deemed unfit for further investigation and given a clinical diagnosis of pleural malignancy and two patients had concurrent fluid cytology consistent with malignancy. Sixteen of these patients had subsequent procedures with six histological and one clinical diagnosis of malignancy made. Of 17 patients that underwent regular follow-up, all either remained well or died within the follow-up period of a non-malignant cause. Including those patients with a clinical diagnosis of malignancy, the overall sensitivity of ultrasound-guided Abrams needle biopsy is 71.4 %, specificity 100 % with a positive predictive value of 100 % and negative predictive value of 72.2 %.

Of the 25 initial Abrams biopsies malignancy, concurrent cytology was available in 23 cases. Thirteen (56.5 %) concurrent samples demonstrated positive cytology confirming malignant cells with 10 (43.5 %) cases failing to yield the diagnosis.

**Fig. 2:**
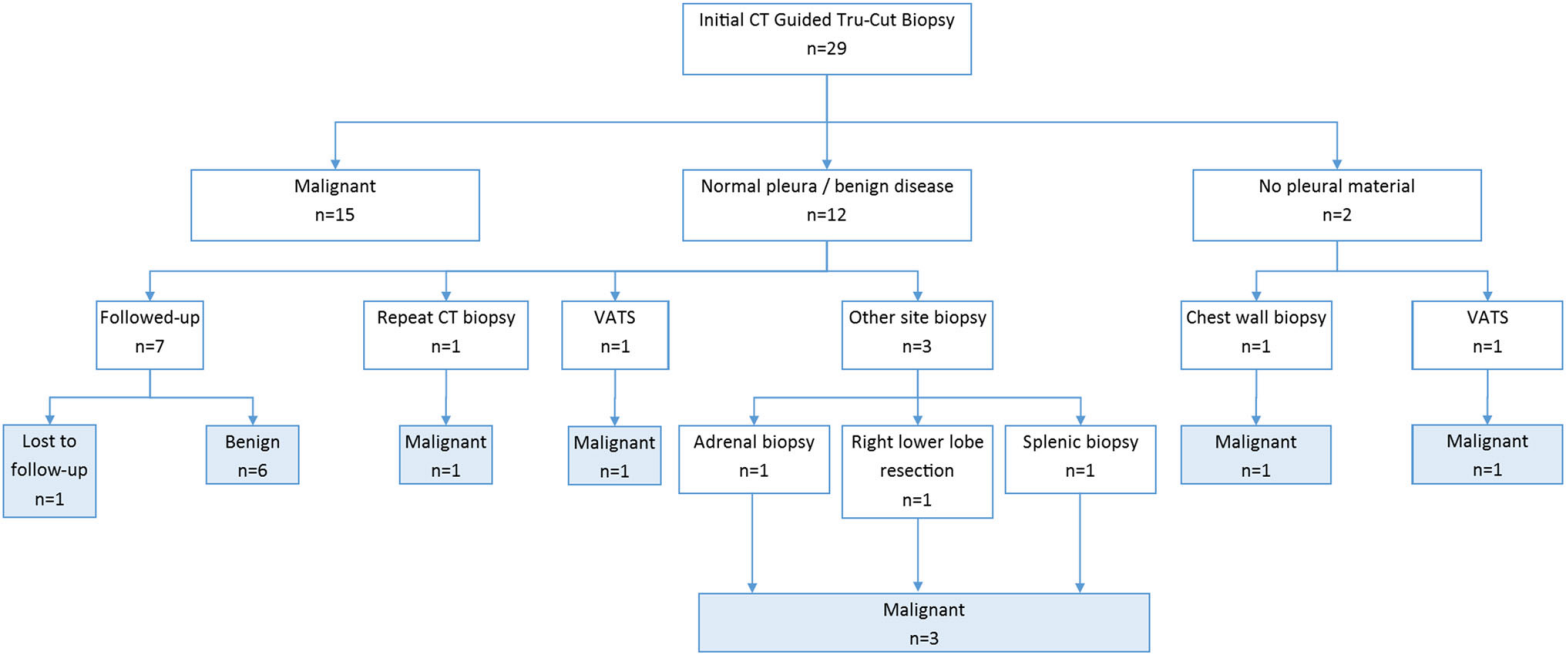
Diagnostic pathway of patients in the CT-guided Tru-Cut group. *Shaded boxes* represent a diagnosis of malignant or benign disease at the end of the follow-up period (*VATS* video-assisted thoracoscopic surgery)

In the CT Tru-Cut group (Fig. 2), malignancy was identified in 15 (68.8 %) patients. Biopsy was non-diagnostic in two (6.3 %) cases. Both patients underwent further sampling demonstrating malignant disease. Twelve (41.4 %) patients had histology consistent with benign disease, five of which underwent a repeat procedure yielding a malignant diagnosis. Four remained well with no diagnosis of malignant disease during the follow-up period. One patient was discharged to a nursing home and lost to further follow-up. Two patients died secondary to a non-malignant cause. Assuming the patient lost to follow-up had a final diagnosis of a benign condition, the sensitivity of CT-guided Tru-Cut biopsy is 75 %, specificity 100 % with a positive predictive value of 100 %, and negative predictive value of 58.3 %. The radiology appearances of pleural disease targeted in the CT biopsy group can be found in supplementary table S2.

### Biopsy Specimen Size

**Fig. 3:**
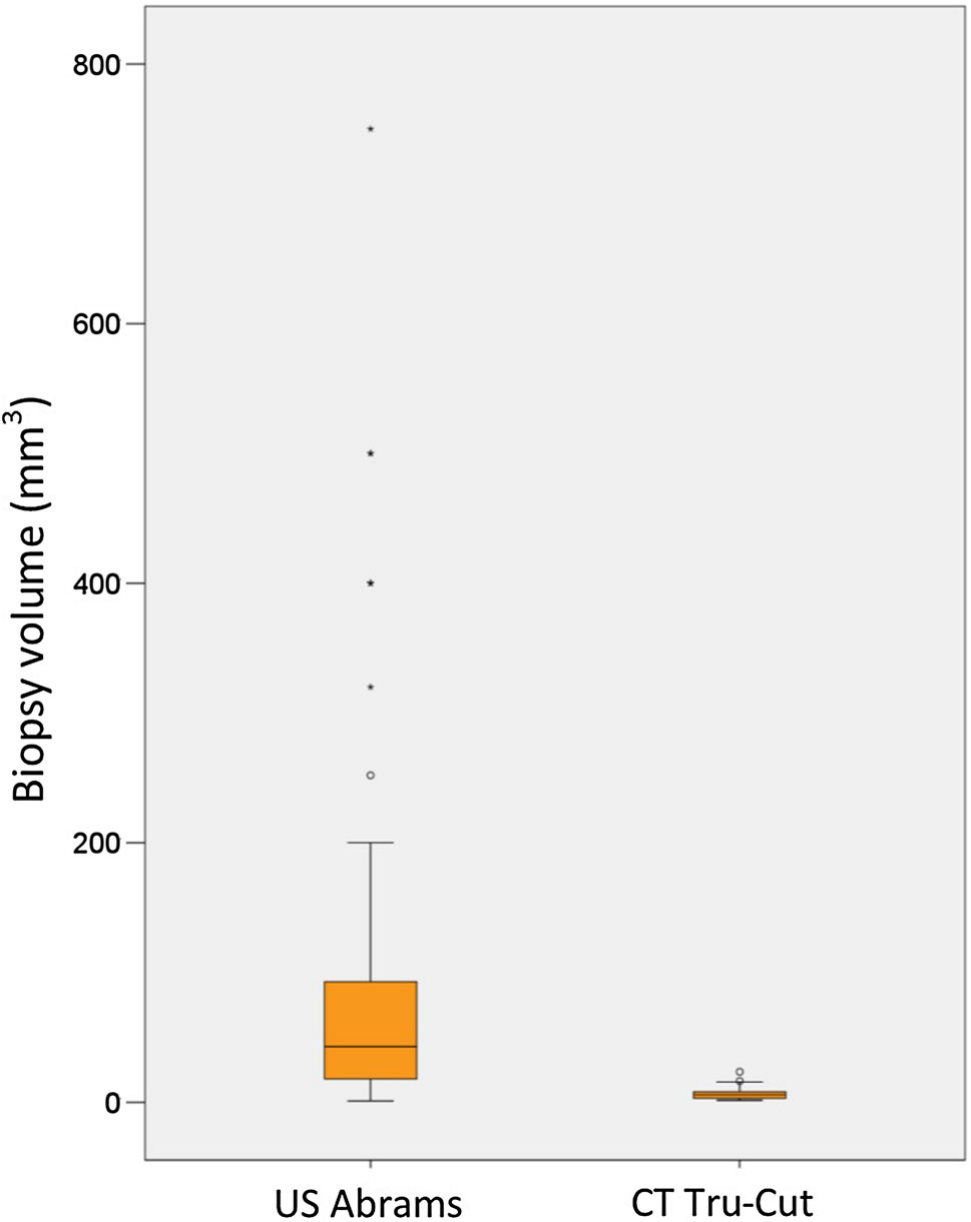
*Box plot* of biopsy volumes in the Abrams and Tru-Cut groups

Data were available on the 86 individual biopsy specimens in the Abrams group vs 56 in the Tru-Cut group (Table 2 and Fig. 3).

**Table 2:** Volume of biopsy samples in the two groups

	US-guided Abrams group	CT-guided Tru-Cut group	
Number of biopsy samples	86	56	
Median volume (mm^3^)	18 (IQR 16–60)	7.1 (IQR 3.1–8.0)	Mann–Whitney *U* *p* < 0.001

## Discussion

Our retrospective evaluation shows that ultrasound-directed Abrams biopsy offers superior sensitivity compared to published data with the conventional “blind” approach [6]. The use of image guidance is the only difference in our series increasing the diagnostic yield versus the published literature. A considerable proportion of those with a positive Abrams biopsy had concurrent negative cytology, suggesting that image-guided biopsies may be useful in patients with negative cytology. It also demonstrates comparable sensitivity to the CT group, the performance of which is slightly poorer than published data where the diagnostic yield is quoted as 87 % [12]. A possible explanation is that in our tertiary centre, patients referred for CT biopsy are highly selected, with the radiological feature of pleural nodularity which may prove challenging to access percutaneously.

Thoracic ultrasound is a useful tool in malignant disease which may allow further visualisation and targeting of disease. One study was able to identify 73 % of malignant effusions on US appearance alone with pleural thickening >10 mm, pleural nodularity and diaphragmatic thickening >7 mm highly suggestive of malignant disease [13]. Ultrasound may also be used with Tru-Cut needle biopsies with the advantage of real-time visualisation (as opposed to US-directed site selection with an Abrams needle). Incorporating ultrasound increases the diagnostic yield in malignancy of the Tru-Cut needle to between 70 and 85.5 % [14, 15] compared to 54 % with a blind approach [16]. With growing US expertise amongst respiratory physicians, it has shown to be safe in the hands of respiratory physicians for lesions greater than 20 mm in diameter.

Biopsy volume was significantly larger in the Abrams group. A possible explanation for this is that the Abrams needle used in our case series is eight gauge versus the commonly used Tru-Cut needle sizes between 16 and 19 gauge used in our institute. Although the volume of tissue is significantly lower in the Tru-Cut group, the real-time image guidance afforded in this technique may allow focused targeting of pleural tissue resulting in a satisfactory diagnostic yield. This is also reflected in a study by Koegelenberg et al. which suggests a comparable yield in pleural malignancy between US-guided Abrams and US Tru-Cut biopsies [17].

These results should be interpreted with caution given significant selection bias in our data. The decision of the biopsy technique employed was taken by the physician in a centre with a dedicated pleural service based on clinical data, point of care ultrasound findings, experience and expertise. Those referred for CT biopsy had either minimal effusions (which would preclude the use of Abrams) or a target area not easily identifiable on ultrasound. Cases are also vetted by radiologists resulting in a narrowly selected group of patients. It is also reasonable to assume that those with disease that is difficult to access percutaneously or no focal pleural abnormality would undergo an alternative diagnostic pathway.

The strength of our study is the 3-year follow-up period, which will reduce the false-negative rate. To our knowledge, there are no published data on long-term follow-up investigating the false-negative rate following image-guided biopsies. It also has real world applicability, especially as the population studied has followed a pathway where the choice of biopsy technique depends heavily on the clinical and radiological picture.

## Conclusion

Image-guided pleural biopsy is useful in the diagnostic workup of a selected group of patients with suspected non-tuberculous pleural disease. A larger prospective trial is needed for a more definitive answer. Although one single technique may not be suitable for all patients, a satisfactory diagnostic yield is achieved if the biopsy method is selected based on clinical and radiological amenability, as well as operator experience and confidence.

## Electronic supplementary material

Below is the link to the electronic supplementary material.

Supplementary material 1 (PDF 119 kb)

Supplementary material 2 (PDF 114 kb)
