# Supplementary material for: Ultrasound-Guided Abrams Pleural Biopsy vs CT-Guided Tru-Cut Pleural Biopsy in Malignant Pleural Disease, a 3-Year Follow-up Study
Source: Lung. 2016 Aug 19;194(6):911–6. doi: 10.1007/s00408-016-9933-9 (PMC5093211; doi:10.1007/s00408-016-9933-9)
Supplement: Supplementary file 1 — Supplementary material 1 (PDF 119 kb) [file 408_2016_9933_MOESM1_ESM.pdf]

Supplementary Table S1: Malignant diagnoses in the two cohorts

	Ultrasound Guided Abrams Group	CT Guided Tru-Cut Group
Non-small cell carcinoma	15	6
Breast carcinoma	5	-
Mesothelioma	6	6
Small cell lung carcinoma	1	1
Thyroid carcinoma	-	1
Malignant melanoma	-	1
Colorectal carcinoma	-	1
Upper GI carcinoma	-	2
Renal cell carcinoma	-	1
Bladder carcinoma	-	1
B Cell Lymphoma	-	1
Cutaneous T Cell Lymphoma	-	1
Mantle cell Lymphoma	1	-
Endometrial	1	-
Epitheloid haemangioendothelioma	1	-
Adenocarcinoma of unknown primary	2	-
Ovarian	1	-
Clinical diagnosis of malignancy	2	0
