# Supplementary material for: Ultrasound-Guided Abrams Pleural Biopsy vs CT-Guided Tru-Cut Pleural Biopsy in Malignant Pleural Disease, a 3-Year Follow-up Study
Source: Lung. 2016 Aug 19;194(6):911–6. doi: 10.1007/s00408-016-9933-9 (PMC5093211; doi:10.1007/s00408-016-9933-9)
Supplement: Supplementary file 2 — Supplementary material 2 (PDF 114 kb) [file 408_2016_9933_MOESM2_ESM.pdf]

Supplementary Table 2: Indications for CT Biopsy

Indication for CT Biopsy	Number
Focal pleural nodule / lesion	19 (65.5%)
Pleural thickening	10 (34.5%)
